# Alcohol use in self-isolation during the COVID-19 pandemic: a cross-sectional survey in Brazil

**DOI:** 10.47626/2237-6089-2021-0337

**Published:** 2023-01-04

**Authors:** Helena F. Moura, Lisia von Diemen, Rugero A. Bulzing, Jacob Meyer, Igor Grabovac, Guillermo F. López-Sánchez, Kabir P. Sadarangani, Mark A. Tully, Lee Smith, Felipe B. Schuch

**Affiliations:** 1 Programa de Pós-Graduação em Psiquiatria e Ciências do Comportamento Departamento de Psiquiatria Universidade Federal do Rio Grande do Sul Porto Alegre RS Brazil Programa de Pós-Graduação em Psiquiatria e Ciências do Comportamento, Departamento de Psiquiatria, Universidade Federal do Rio Grande do Sul (UFRGS), Porto Alegre, RS, Brazil.; 2 Departamento de Clínica Médica Faculdade de Medicina Universidade de Brasília Brasília DF Brazil Departamento de Clínica Médica, Faculdade de Medicina, Universidade de Brasília, Brasília, DF, Brazil.; 3 Departamento de Métodos e Técnicas Desportivas Universidade Federal de Santa Maria Santa Maria RS Brazil Departamento de Métodos e Técnicas Desportivas, Universidade Federal de Santa Maria (UFSM), Santa Maria, RS, Brazil.; 4 Department of Kinesiology Iowa State University Ames IA USA Department of Kinesiology, Iowa State University, Ames, IA, USA.; 5 Department of Social and Preventive Medicine Centre for Public Health Medical University of Vienna Vienna Austria Department of Social and Preventive Medicine, Centre for Public Health, Medical University of Vienna, Vienna, Austria.; 6 Department of Public Health Sciences School of Medicine University of Murcia Murcia Spain Division of Preventive Medicine and Public Health, Department of Public Health Sciences, School of Medicine, University of Murcia, Murcia, Spain.; 7 Universidad Autónoma de Chile Santiago Chile Universidad Autónoma de Chile, Santiago, Chile.; 8 Escuela de Kinesiología Facultad de Salud y Odontología Universidad Diego Portales Santiago Chile Escuela de Kinesiología, Facultad de Salud y Odontología, Universidad Diego Portales, Santiago, Chile.; 9 School of Medicine Ulster University Belfast UK School of Medicine, Ulster University, Belfast, UK.; 10 Centre for Health Anglia Ruskin University Cambridge UK Centre for Health, Performance and Wellbeing, Anglia Ruskin University, Cambridge, UK.

**Keywords:** Alcohol, COVID-19, depression, anxiety

## Abstract

**Objectives:**

To assess alcohol use and perceived change in alcohol consumption (before and during the pandemic) in Brazilians during the COVID-19 pandemic, their correlates, and their associations with depressive, anxiety and co-occurring depressive and anxiety symptoms (D&A).

**Methods:**

This is a cross-sectional study comprising 992 individuals in self-isolation. A self-report questionnaire was used to assess whether participants were drinking during self-isolation and whether they changed their drinking behavior (drinking less, more, or no change) from before to during the pandemic. D&A symptoms were assessed using the Beck Depression and Anxiety Inventories (BDI and BAI).

**Results:**

A total of 68.5% of participants reported alcohol consumption during the pandemic, and 22.7% of these reported increased alcohol use. Smoking was positively associated with alcohol consumption during the pandemic. Alcohol consumption was associated with anxiety (OR = 1.40, 95%CI 1.06-1.85, p < 0.01) and D&A (OR = 1.38, 95%CI 1.02-1.87, p = 0.033) symptoms.

**Conclusions:**

Drinking during self-isolation was prevalent and was associated with risk factors for alcohol use disorders. The long-term effects of high drinking rates and increased consumption should be proactively monitored and assessed.

## Introduction

The COVID-19 pandemic has caused an unprecedented global health crisis with consequences stemming not only from the morbidity and mortality of the virus, but also from prevention strategies.^1^ Social distancing measures have been implemented worldwide to reduce infection transmission, generating considerable disruption in people’s routines and psychological well-being.^2^ In this scenario, changes in alcohol consumption are expected and may have consequences for pandemic management and the population’s mental health.^3^

Studies from previous global crises showed no changes in alcohol use in the overall population.^4 , 5^ Notwithstanding, this appearance of stability occurred at the expense of decreased use among some populations while more vulnerable subgroups increased their use of alcohol.^4^ Therefore, changes were observed in the demographic profile of problem drinkers. Additionally, countries can be affected differently. After the 2008 Great Recession, problem drinking increased significantly more among less educated men in the US, whereas in Spain highly educated women were more affected.^4 , 6^ Overall, anxiety and depressive symptoms mediated drinking behavior.^4^

So far, studies conducted during the COVID-19 pandemic have shown both increases and decreases in alcohol use, which can vary according to environmental, societal, and individual factors, including psychological distress.^4 , 7 - 9^ Environmental and societal factors, such as lack of social events and absence of peer pressure to drink have been associated with decreased use,^10 , 11^ whereas increased use has been associated with some individual factors, such as stress and loneliness.^7 - 13^ Additionally, higher age, higher income, and unemployment have also been associated with increased drinking.^9 , 13^

A study conducted by The Pan-American Health Organization (PAHO) identified a reduction in heavy drinking, except among individuals with anxiety symptoms, in which this behavior increased.^14^ However, their results comprised all countries from Latin America and the Caribbean and, except for an early descriptive data analysis,^12^ little is known about the Brazilian population’s drinking behavior during self-isolation. Therefore, our aims were to:1) assess alcohol use and changes during self-isolation in Brazil; 2) assess the demographic correlates of alcohol use and changes in use; and 3) assess the associations between alcohol use and changes and depressive, anxiety, and co-occurring depressive and anxiety symptoms. We hypothesize that drinking behavior would be associated with socioeconomic status, sex, age, clinical or psychiatric morbidity, and psychiatric symptoms.

## Methods

### Recruiting and inclusion criteria

The study data are derived from an online survey and the methods used have been described elsewhere.^15^ Individuals (≥ 18 years) living in Brazil and in self-isolation (staying at home and leaving home for essential activities only, such as buying food or medicine or visiting a physician) due to the COVID-19 pandemic, who agreed to participate in the survey were eligible. In Brazil, social distancing measures started in March 2020 and were heterogenous across states and cities and the decision to self-isolate was voluntary. Data was collected between April 5th and May 11th, 2020.

### Alcohol consumption use and increase during the pandemic (outcome)

Alcohol consumption was assessed with two questions: “Do you drink alcoholic beverages?’’ and, “Now that you are in self-isolation, do you consider that: you are drinking more, drinking less, or drinking the same amount?”

### Demographic correlates

Demographic data were collected, as well as days in self-isolation, current smoking, and self-reported previous diagnosis of chronic physical diseases and psychiatric disorders.

### Mental health assessment

Depressive symptoms were assessed with the Beck Depression Inventory (BDI),^16^ anxiety symptoms were assessed with the Beck Anxiety Inventory (BAI).^17^ The cutoffs used were: BDI > 9 = prevalent depressive symptoms,^16^ BAI > 7 = prevalent anxiety symptoms,^17^ and BDI > 9 + BAI > 7 = prevalent co-occurring depressive and anxiety (D&A) symptoms.

### Statistical analysis

Descriptive data for continuous variables were expressed as mean (standard deviation) or median and interquartile range (IQR). Categorical variables were expressed as absolute frequencies (%). Logistic regression models were used to evaluate the correlates of alcohol consumption, increase in alcohol consumption (before and during the self-isolation period), and the associations between alcohol consumption (yes vs. no) or increase in alcohol use (using “drinking the same amount” plus “drinking less” as the reference group) and symptoms of depression, anxiety, or D&A. The models tested were adjusted for age, sex, ethnicity, marital status, employment, family income, and number of household inhabitants. Results from the logistic regression models were presented as odds ratios (ORs). Statistical significance was set at p < 0.05. The statistical analysis was performed with SPSS version 22.0 (IBM Corporation).

### Ethical considerations

The study was approved by the Federal University of Santa Maria Research Ethics Committee and by the National Research Ethics Commission (CONEP; 30.244.620.1.0000.5346).

## Results

Demographic data and other descriptive analyses are detailed in Table 1 .

**Table 1:** Sample characteristics: overall and stratified by alcohol consumption

Category	Overall N = 992* (%)	Alcohol consumption		Change in alcohol consumption^**†**^		

		Yes N = 683 (68.9%)	No N = 309 (31.1%)	Less N = 222 (32.5%)	More N = 155 (22.7%)	No change N = 305 (44.7%)
Sex						
Women	713 (72.2)	478 (67)	235 (33.0)	140 (29.3)	114 (23.8)	224 (49.6)
Men	274 (27.8)	201 (73.4)	73 (26.6)	80 (40)	40 (20)	80 (40)
Age						
18-24 years	140 (14.1)	103 (73.6)	37 (26.4)	51 (49,5)	15 (14.6)	37 (35,9)
25-34 years	381 (38.4)	267 (70.1)	114 (29.9)	89 (33,3)	66 (24.7)	112 (41,9)
35-44 years	248 (25.0)	177 (71.4)	71 (28.6)	51 (28.8)	49 (27.7)	77 (43.5)
45-54 years	121 (12.2)	79 (65.3)	42 (34.7)	20 (25.3)	18 (22.8)	41 (51.9)
≥ 55 years	102 (10.3)	57 (55.9)	45 (44.1)	11 (19.6)	7 (12.5)	38 (67.9)
Ethnicity						
White	756 (76.4)	533 (70.5)	223 (29.5)	161 (30.3)	123 (23.1)	248 (46.6)
Non-White	233 (23.6)	147 (63.1)	86 (36.9)	60 (40.8)	32 (21.8)	55 (37.4)
Marital status						
Married	420 (42.8)	284 (67.6)	136 (32.4)	70 (24.7)	68 (24.0)	145 (51.2)
No partner	562 (57.2)	393 (69.9)	169 (30.1)	149 (37.9)	87 (22.1)	157 (39.9)
Employment^‡^						
Regular	551 (55.6)	368 (66.8)	183 (33.2)	106 (28.9)	90 (24.5)	171 (46.6)
Unemployed	45 (4.5)	27 (60.0)	18 (40.0)	11 (40.7)	4 (14.8)	12 (44.4)
Irregular	395 (39.9)	287 (72.7)	108 (27.3)	104 (36.2)	61 (21.3)	122 (42.5)
Monthly household income^§^						
< US$ 231	34 (3.4)	22 (64.7)	12 (35.3)	9 (40,9)	7 (31.8)	6 (27.3)
US$ 232 - US$ 1,595	518 (52.3)	343 (66.2)	175 (33.8)	135 (39.4)	65 (19.0)	143 (41.7)
US$ 1,596 - US$ 2,079	150 (15.1)	109 (72.7)	41 (27.3)	30 (27.5)	28 (25.7)	51 (46.8)
≥ US$ 2,080	289 (29.2)	208 (72.0)	81 (28.0)	47 (22.7)	55 (26.6)	105 (50.7)
Household inhabitants						
1	143 (14.4)	101 (71.1)	41 (28.9)	32 (301.7)	26 (25.7)	43 (42.6)
2	335 (33.7)	236 (70.9)	97 (29.1)	66 (28)	61 (25.8)	109 (46.2)
3	267 (26.8)	179 (67.3)	87 (32.7)	62 (34.6)	35 (19.6)	82 (45.8)
4	185 (18.6)	127 (68.6)	58 (31.4)	45 (35.7)	26 (20.6)	55 (43.7)
5 or more	65 (6.5)	39 (60.0)	26 (40.0)	16 (41)	7 (17.9)	16 (41)
Current smoking						
No	941 (94.9)	643 (68.3)	298 (31.7)	212 (33)	141 (22)	289 (45)
Yes	51 (5.1)	40 (78.4)	11 (21.6)	10 (25)	14 (35)	16 (40)
Days in self-isolation						
Median (IQR)	28.00 (8.0)	28.00 (8.0)	28.00 (9.0)	28.00 (7.75)	25.00 (10.0)	28.00 (6.0)
Self-reported previous diagnosis of physical conditions						
No	61 (6.1)	44 (72.1)	17 (27.9)	17 (38,6)	13 (29.5)	14 (31.8)
Yes	931 (93.9)	639 (68.6)	292 (31.4)	205 (32,1)	142 (22.3)	291 (45.6)
Self-reported previous diagnosis of psychiatric conditions						
No	595 (60.0)	420 (70.6)	175 (29.4)	144 (27.7)	93 (17.9)	282 (54.3)
Yes	397 (40.0)	263 (66.2)	134 (33.8)	99 (37,6)	62 (23.6)	102 (38.8)
BAI						
Median (IQR)	6.00 (11.0)	7.00 (11.0)	5.00 (10.0)	7.00 (11.0)	7.00 (10.0)	6.00 (9.0)
BDI						
Median (IQR)	9.00 (10.0)	9.00 (9.0)	8.00 (10.0)	10.00 (9.0)	9.00 (9.0)	8.00 (9.0)

BAI = Beck Anxiety Inventory; BDI = Beck Depression Inventory; IQR = interquartile range.

* Total sample size with available data. Number of cases can vary for each variable due to missing cases (minimum = 927);

^†^ Includes only those who reported alcohol consumption;

^‡^ The regular group includes those with regular incomes: employed, retired, and military; the irregular/unknown group includes those with irregular incomes: students, the self-employed, and housewives.

^**§**^ Approximate values based on the exchange rate on January 9, 2021.

### Prevalence of alcohol consumption during the pandemic and its correlates

A total of 683 (68.9%) participants reported alcohol consumption whilst self-isolating (during self-isolation). Smoking was significantly associated with higher alcohol consumption (OR = 2.1, 95%CI 1.05-4.41, p = 0.03). Being ≥ 55 versus 18-24 years old (OR = 0.34, 95%CI 0.18-0.66, p < 0.01) and having a monthly household income ranging from US$ 232 - US$ 1,595 (OR = 0.53, 95%CI 0.39-0.83, p < 0.01) were negatively associated with alcohol consumption during self-isolation (see Table 2 ).

**Table 2:** Correlates of alcohol consumption during self-isolation

	B	SE	Sig.	95%CI for OR		

				OR	Lower	Upper
Men vs. women	0.300	0.166	0.071	1.350	0.975	1.868
Ethnicity						
Non-white vs. white	-0.267	0.166	0.109	0.766	0.552	1.061
Marital status						
No partner vs. married	0.070	0.176	0.693	1.072	0.759	1.514
Monthly household income						
< US$ 231 vs. ≥ US$ 2,080	-0.808	0.448	0.071	0.446	0.185	1.072
US$ 232- US$ 1,595 vs. ≥ US$ 2,080	-0.562	0.191	**0.003**	0.570	0.392	0.829
US$ 1,596- US$ 2,079 vs. ≥ US$ 2,080	-0.197	0.237	0.406	0.822	0.517	1.306
Household inhabitants						
1 vs. 5 or more	0.649	0.345	0.060	1.913	0.974	3.759
2 vs. 5 or more	0.473	0.299	0.113	1.605	0.894	2.884
3 vs. 5 or more	0.244	0.298	0.414	1.276	0.711	2.290
4 vs. 5 or more	0.271	0.312	0.386	1.311	0.711	2.419
Smoking vs. non-smoking	0.771	0.365	**0.034**	2.163	1.058	4.419
Lifetime psychiatric disorder						
Yes vs. no	0.178	0.148	0.228	1.195	0.895	1.596
Lifetime physical disorder						
Yes vs. no	0.000	0.314	0.999	1.000	0.540	1.850
Age						
25-34 vs. 18-24 years	-0.173	0.246	0.482	0.841	0.519	1.362
35-44 vs. 18-24 years	-0.178	0.290	0.540	0.837	0.474	1.477
45-54 vs. 18-24 years	-0.453	0.322	0.160	0.636	0.338	1.196
≥ 55 vs. 18-24 years	-1.063	0.334	**0.001**	0.345	0.180	0.664
Employment*						
Non-regular/unknown vs. regular	0.334	0.176	0.057	1.397	0.990	1.972
Unemployed vs. regular	-0.107	0.346	0.757	0.898	0.456	1.771

95%CI = 95% confidence interval; OR = odds ratio; SE = standard error.

Bold type denotes significant *p* -values.

* The regular group includes those with regular incomes: employed, retired, and military; the irregular/unknown group includes those with irregular incomes: students, the self-employed, and housewives.

Odds of those who reported alcohol consumption versus those who reported no alcohol consumption (reference).

### Changes in alcohol consumption in self-isolation (before and during) and correlates

A total of 155 (22.7%) participants reported an increase in alcohol consumption, whereas 265 (32.5%) reported a reduction. Smoking was associated with an increase in alcohol consumption (OR = 2.4, 95%CI 1.23-4.76, p = 0.01) (see Table 3 ). A household income ranging from US$ 232 - US$ 1,595 was associated with a lower risk of an increase (OR = 0.54, 95%CI 0.34-0.86, p < 0.01) in alcohol consumption.

**Table 3:** Correlates of more alcohol consumption in self-isolation (from pre to during)

	B	SE	Sig.	95%CI for OR		
				OR	Lower	Upper
Men vs. women	-0.279	0.211	0.187	0.757	0.500	1.145
Ethnicity						
Non-white vs. white	-0.033	0.226	0.883	0.967	0.621	1.507
Marital status						
No partner vs. married	0.026	0.226	0.909	1.026	0.659	1.597
Monthly household income						
< US$ 231 vs. ≥ US$ 2,080	0.336	0.512	0.511	1.399	0.513	3.815
US$ 232- US$ 1,595 vs. ≥ US$ 2,080	-0.617	0.236	**0.009**	0.539	0.340	0.856
US$ 1,596 - US$ 2,079 vs. ≥ US$ 2,080	-0.239	0.275	0.385	0.787	0.459	1.350
Household inhabitants						
1 vs. 5 or more	0.674	0.490	0.169	1.963	0.752	5.125
2 vs. 5 or more	0.657	0.442	0.138	1.928	0.810	4.590
3 vs. 5 or more	0.148	0.455	0.745	1.160	0.475	2.831
4 vs. 5 or more	0.341	0.468	0.466	1.407	0.562	3.521
Smoking vs. non-smoking	0.881	0.346	**0.011**	2.414	1.226	4.757
Lifetime psychiatric disorder						
Yes vs. no	-0.032	0.189	0.867	0.969	0.669	1.403
Lifetime physical condition						
Yes vs. no	0.321	0.352	0.362	1.379	0.691	2.750
Age						
25-34 vs. 18-24 years	0.522	0.336	0.120	1.685	0.873	3.255
35-44 vs. 18-24 years	0.591	0.380	0.119	1.806	0.858	3.800
45-54 vs. 18-24 years	0.507	0.428	0.236	1.660	0.718	3.838
≥ 55 vs. 18-24 years	-0.495	0.518	0.339	0.609	0.221	1.684
Employment*						
Irregular/unknown vs. regular	0.084	0.218	0.701	1.087	0.709	1.666
Unemployed vs. regular	-0.676	0.537	0.208	0.509	0.178	1.456

95%CI = 95% confidence interval; OR = odds ratio; SE = standard error.

Bold type denotes significant *p* -values.

* The regular group includes those with regular incomes: employed, retired, and military; the irregular/unknown group includes those with irregular incomes: students, the self-employed, and housewives.

Odds of those who reported consuming more alcohol consumption versus those who reported less consumption or no change in alcohol consumption (reference).

### Associations between alcohol consumption and changes in alcohol consumption and depressive, anxiety, and co-occurring depressive and anxiety symptoms

Alcohol consumption during self-isolation was associated with anxiety (OR = 1.40, 95%CI 1.06-1.85, p < 0.01) and D&A symptoms (OR = 1.38, 95%CI 1.02-1.87, p = 0.033) even after adjusting for confounding factors (OR = 1.52, 95%CI 1.13-2.05, p = 0.006 and OR = 1.48, 95%CI 1.07-2.04, p = 0.018, respectively) in logistic regression models. We found no association between psychiatric symptoms and drinking more ( Table 4 ).

**Table 4:** Cross-sectional logistic associations between prevalent mental health symptoms and alcohol use during the COVID-19 pandemic in 2020 in Brazil

	Crude				Adj. 1				Adj. 2			
	OR	95%CI		p	OR	95%CI		p	OR	95%CI		p
Alcohol use*												
Depression	1.16	0.88	1.53	0.280	1.14	0.85	1.52	0.149	1.25	0.92	1.68	0.149
Anxiety	**1.40**	**1.06**	**1.85**	**0.016**	**1.40**	**1.05**	**1.87**	**0.020**	**1.52**	**1.13**	**2.05**	**0.006**
Depression and anxiety	**1.38**	**1.02**	**1.87**	**0.033**	**1.35**	0.99	1.84	0.057	**1.48**	**1.07**	**2.04**	**0.018**
More alcohol use^†^												
Depression	1.18	0.84	1.68	0.341	1.19	0.83	1.71	0.351	1.32	0.90	1.93	0.149
Anxiety	1.11	0.78	1.56	0.572	1.10	0.77	1.58	0.596	1.23	0.85	1.79	0.276
Depression and anxiety	1.18	0.82	1.69	0.373	1.19	0.82	1.73	0.143	1.34	0.90	1.99	0.143

95%CI = confidence interval; OR = odds ratio.

* Odds of those who reported alcohol consumption versus those who reported no alcohol consumption (reference; N = 976) having prevalent depressive symptoms (BDI > 9), anxiety symptoms (BAI > 7), or co-occurring depression and anxiety symptoms (BDI > 9 & BAI > 7).

^†^ Odds of those who reported more alcohol consumption versus those who reported reduced consumption or no change in alcohol consumption (reference; N = 860) having prevalent depressive symptoms (BDI > 9), anxiety symptoms (BAI > 7), or co-occurring depression and anxiety symptoms (BDI > 9 & BAI > 7).

The models shown are: crude, with no adjustments; Adjusted 1 (Adj. 1). adjusted for age and sex; and Adjusted 2 (Adj. 2). adjusted for age, sex, ethnicity, marital status, employment, family income, and number of household inhabitants.

## Discussion

We found that 68.5% of our participants reported drinking during the pandemic and that 22.7% of those who did so reported increased drinking while 32.5% reported a decrease in alcohol consumption. Smoking was positively associated with both drinking and increased drinking. Middle-aged adults were at lower risk of drinking, whereas average income was associated with lower risk of both drinking and increased drinking. Importantly, drinking was associated with anxiety and co-occurring depression and anxiety.

Garcia-Cerde et al. found similar prevalence of drinking during quarantine in Latin American and Caribbean countries.^14^ Their pre-pandemic prevalence of drinkers and binge drinking were also similar to those found in Brazil. More specifically, the prevalence of alcohol drinkers in Brazil was 66.4%, 38.4% of whom reported binge drinking.^18^ Additionally, 20% of binge drinkers consume 56% of all the country’s alcoholic beverages.^19^ The distribution of drinkers between age groups and sex in our sample was also similar to that found in a national survey, except that we found a higher prevalence among women (59% vs. 67%).^18^ Considering that the alcohol consumption profile of our sample resembled that of the Brazilian or Latin American and Caribbean populations, the prevalence of those who drink or increased drinking during isolation is worrying. Alcohol consumption is related to a higher risk of infection and complications from respiratory viruses, and increases in interpersonal violence, reasons why the World Health Organization (WHO) recommended restrictions to its access during lock down.^20^

The COVID-19 pandemic has brought financial insecurity and, consequently, a great psychological burden, especially to the working-age population. Previous studies of economic crises and alcohol use have shown that people with lower income reduce their drinking due to loss of resources, whereas the opposite occurs among those with higher incomes, in which the economic factor may be overlaid by drinking as a coping strategy.^21^ In fact, higher income was associated with heavy drinking episodes during the pandemic in the PAHO study.^14^ However, we found that middle, but not low income was associated with a lower risk of alcohol use before and during the pandemic. This result could have been a consequence of the small number of low-income individuals in our sample, although the availability of very cheap alcoholic beverages in Brazil should not be ignored. Since alcohol consumption increases the risk of infection and complications of COVID-19,^3^ reducing consumption among the poorest could mitigate the damage to this population, which has been most severely affected by the pandemic.^22^

Additionally, our middle-class sample was comprised predominantly of young adults. They may have a lower risk of drinking due to parenting responsibilities, starting a working career, or because they prefer to drink in social settings. Nevertheless, we did not find an association between young adulthood and drinking before or during self-isolation.

Notably, anxiety and co-occurring D&A symptoms were associated with drinking even after controlling for other covariates. These psychiatric comorbidities have been associated with higher risk for alcohol use disorders (AUD), faster progression of AUD, and a greater need for hospitalization for both AUD and comorbid psychiatric disorders.^23 , 24^

Quarantine, depression, and drinking to cope were independently and positively associated with AUD symptoms three years after the SARS epidemic in China.^25^ Psychological distress due to social isolation, fear of contamination, grief, and financial insecurity have been associated with quarantines^2 , 26^ and may lead to alcohol use as a coping strategy.^3^ In that sense, drinking to self-medicate negative emotions may have prevented some individuals from reducing their drinking during this period of reduced environmental stimuli (i.e. public places and events associated with drinking). However, psychiatric symptoms can also be a consequence of alcohol use. Neurobiological studies have found overlaps in the neural circuitry of both substance use disorders and depression and anxiety disorders.^27^ Anyhow, because increases in anxiety and depressive symptoms have been associated with the current pandemic,^2 , 28^ careful assessment of alcohol use among individuals with these symptoms is imperative.

We found no association between psychiatric symptoms and increased alcohol use. One possible explanation is that individuals with anxiety and D&A symptoms already had high levels of alcohol use before the pandemic.

Not surprisingly, we found that smoking was associated with both alcohol use and increased use. The combination of nicotine and alcohol is highly comorbid and is associated with cross-reinforcement in the reward system as well as with cross-tolerance, enhancing the risk of hazardous drinking.^23 , 29^

Our study has some limitations. First, because we used secondary data from another study,^15^ we did not use validated scales for AUD. Also, alcohol consumption or even increasing alcohol consumption does not *per se* mean hazardous drinking. However, our findings of drinking being associated with known risk factors for AUD is worrying and should encourage further investigation. Moreover, we found that those individuals who had been drinking were more likely to present anxiety and co-occurring D&A, which indicates a need to target individuals with psychiatric symptoms. Another limitation is that the study sample is not representative of the Brazilian population. Women were overrepresented and most participants were from the South and Southeast regions of Brazil. Additionally, low-income populations and smokers were underrepresented. Finally, this is a cross-sectional study and causal relationships cannot be inferred, and memory bias might play a role.

In conclusion, drinking during self-isolation was prevalent, varied across demographic subgroups, and was associated with higher risk of smoking and anxiety and D&A symptoms. The long-term effects of increased consumption can have implications for public health. Therefore, it should be monitored and assessed, since both smoking and psychiatric symptoms were associated with AUD.
